# CVD transfer-free graphene for sensing applications

**DOI:** 10.3762/bjnano.8.102

**Published:** 2017-05-08

**Authors:** Chiara Schiattarella, Sten Vollebregt, Tiziana Polichetti, Brigida Alfano, Ettore Massera, Maria Lucia Miglietta, Girolamo Di Francia, Pasqualina Maria Sarro

**Affiliations:** 1 University of Naples “Federico II”, Department of Physics “E. Pancini”, Naples, Italy; 2 Delft University of Technology, Department of Microelectronics, Delft, The Netherlands; 3 ENEA C.R. Piazzale Enrico Fermi, 1, 80055 Portici (Naples), Italy

**Keywords:** ammonia, chemiresistors, CMOS-compatible process, graphene, nitrogen dioxide, transfer-free growth

## Abstract

The sp^2^ carbon-based allotropes have been extensively exploited for the realization of gas sensors in the recent years because of their high conductivity and large specific surface area. A study on graphene that was synthetized by means of a novel transfer-free fabrication approach and is employed as sensing material is herein presented. Multilayer graphene was deposited by chemical vapour deposition (CVD) mediated by CMOS-compatible Mo. The utilized technique takes advantage of the absence of damage or contamination of the synthesized graphene, because there is no need for the transfer onto a substrate. Moreover, a proper pre-patterning of the Mo catalyst allows one to obtain graphene films with different shapes and dimensions. The sensing properties of the material have been investigated by exposing the devices to NO_2_, NH_3_ and CO, which have been selected because they are well-known hazardous substances. The concentration ranges have been chosen according to the conventional monitoring of these gases. The measurements have been carried out in humid N_2_ environment, setting the flow rate at 500 sccm, the temperature at 25 °C and the relative humidity (RH) at 50%. An increase of the conductance response has been recorded upon exposure towards NO_2_, whereas a decrease of the signal has been detected towards NH_3_. The material appears totally insensitive towards CO. Finally, the sensing selectivity has been proven by evaluating and comparing the degree of adsorption and the interaction energies for NO_2_ and NH_3_ on graphene. The direct-growth approach for the synthesis of graphene opens a promising path towards diverse applicative scenarios, including the straightforward integration in electronic devices.

## Introduction

Due to its extraordinary electronic, chemical, mechanical, thermal and optical properties, graphene has been defined as the “wonder material” of the 21st century with plentiful applications in several fields such as energy storage, biomedical, electronics [1]. Among these applications, one of the most promising is likely that in the field of gas sensors, where the main requirements, namely high specific surface-to-volume ratio, high mobility and low electrical noise, are all precisely concentrated in this material. As it has been reported by Varghese et al. in a recent review on chemical sensors, the interest of the scientific community towards graphene for sensing applications is continuously growing, as testified by the increasing number of publications dedicated to graphene-based sensors [2]. The gas sensor devices presented in literature are mostly based on pristine graphene, graphene oxide (GO) and reduced graphene oxide (rGO). Many approaches for the fabrication of such materials, including CVD, mechanical, chemical and electrochemical exfoliation can be employed. Each provides a sensing layer with a pronounced specificity towards a particular analyte [3–6]. However, the issue of selectivity is far away from being solved. A possible solution could be represented by resorting to the design of an array of sensors that can discriminate between analytes by comparing and analysing responses from multiple devices [7].

Another issue that needs to be addressed is the problem of slow and incomplete recovery in the operative regime of standard temperature and pressure, which can be solved by providing the sensors with appropriate electronics, able to perform data analysis [8] or a refresh of the device through UV illumination or thermal annealing [9–13].

All that said, the development of new approaches enabling the direct growth of large-area graphene layers on arbitrary insulating substrates, compatible with conventional Si technology, is of crucial importance [14–15]. A possible choice for the synthesis of large-area graphene is the metal-assisted hydrocarbon dissociation and/or the deposition method from solid carbon sources. In both cases, after the growth the graphene film needs to be transferred onto other substrates for the fabrication of devices [16–19]. A step forward in the direction of the transfer-free synthesis has been made by Kwak et al. [20] who proposed a diffusion-assisted synthesis method for uniform and controllable deposition of graphene films with micrometre-sized grains at a Ni/SiO_2_ interface. More recently, Lukosius et al. have shown the successful growth of a graphene layer underneath Ni bars on insulating SiO_2_ layers, so avoiding the metal contamination problems and complexity associated to graphene transfer [21].

In the present work, we show the results of the sensing performances of conductometric devices in which multilayer graphene films have been directly synthesized on insulating SiO_2_ substrates by means of a transfer-free CVD method mediated by Mo. The pre-patterning of the Mo layer allows the graphene to be shaped in the desired form by means of standard lithography techniques, following a completely Si-technology-compatible approach. Chemiresistive devices have been realized by shaping the sensing layer in form of rectangular strips.

In order to probe the gas-sensing capabilities of the fabricated devices, three standard pollutants have been chosen, namely NO_2_, NH_3_ and CO. In particular, NO_2_ and NH_3_ are known to exhibit an electron acceptor- and donor-like nature respectively, as extensively shown in the literature [13,22–23]. The chosen concentration ranges are the same than those monitored in conventional contexts.

Finally, the sensing specificity of the material has been probed by evaluating and comparing the degree of adsorption and the interaction energies for NO_2_ and NH_3_.

## Results and Discussion

### Materials characterization

The material, prepared according to the procedure discussed in detail in the Experimental section, has been morphologically and structurally characterized in a previous work [24], from which it emerged that the average number of layers was about 20. The material showed to be free from metal impurities after Mo removal and not damaged by the lift-off process. The quality of the graphene film has been monitored via Raman spectroscopy throughout the whole fabrication process: after CVD growth, after Mo etching and after lift-off. The absence of contamination has then been attested by EDX analysis.

**Figure 1:**
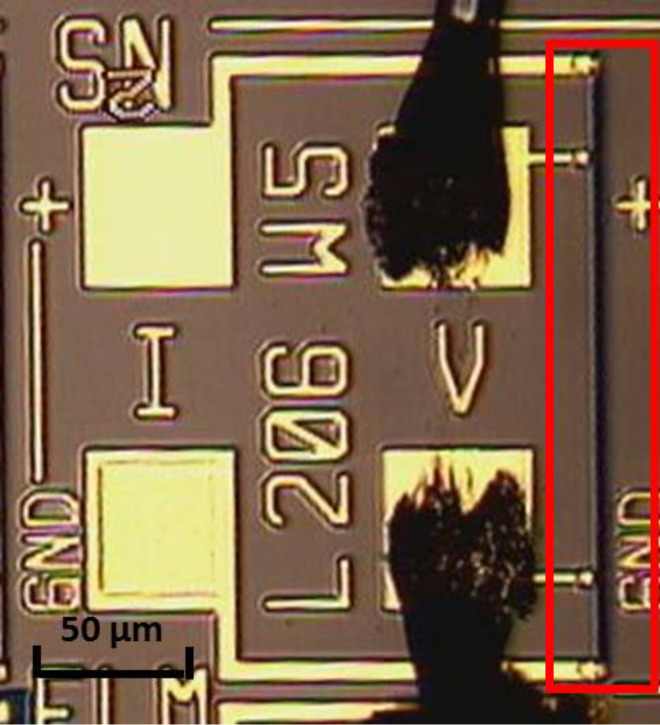
Optical micrograph of one of the CVD graphene-based chemiresistive devices. The graphene strip is highlighted in red. The length *l* and width *w* of the sensing strip (in µm) have also been indicated and patterned between the electrodes.

In Figure 1 a representative optical micrograph of the fabricated devices is reported. The graphene layer (dark strip highlighted in red) and gold contacts with wire-bonds are clearly visible. As can be seen, accurately defined multilayer graphene patterns down to micrometre-size can be obtained using this method. From an electrical point of view, the fabrication technique has proven to be quite repeatable and uniform, since devices with the same geometry either coming from different wafers or within the same one had the same base resistance. Furthermore, the graphene exhibited a p-type electrical characteristic.

**Figure 2:**
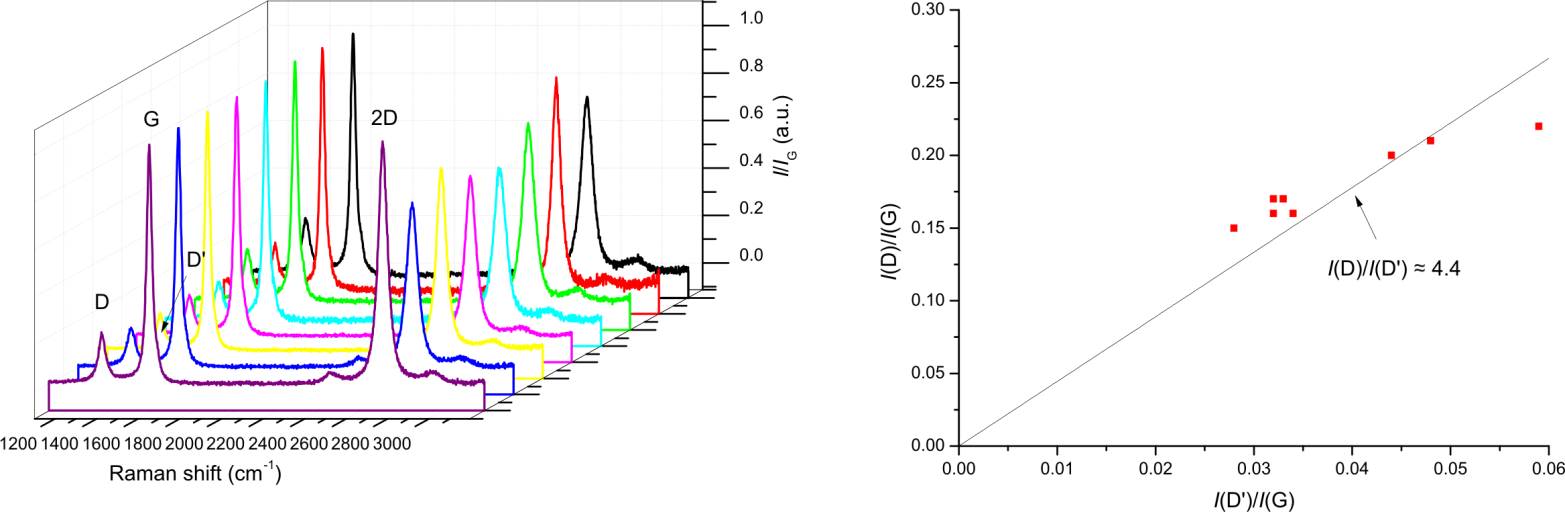
(Left): Raman spectra acquired in different points of the graphene film. (Right): Plot of *I*(D)/*I*(G) vs *I*(D′)/*I*(G) referred to the same spectra.

Starting from this premise, we have further deepened the analysis of the material by performing a Raman analysis in several different sites of the graphene film. As can be seen in Figure 2, the spectra display a fair uniformity. In particular, the arising of the D (ca. 1350 cm^−1^) and D′ (ca. 1600 cm^−1^) bands is related to presence of defects and in particular the D intensity, roughly 5-fold lower than that of the G peak, attests a low density of defects. This evidence confirms the non-degradation of the material even during ageing.

The analysis of the *I*(D)/*I*(D′) ratio allows one to discriminate the type of defects in the film. Indeed, as reported by Eckmann et al., the value of the slope of the *I*(D)/*I*(G) vs *I*(D′)/*I*(G) curve, namely *I*(D)/*I*(D′), can be related to the preponderant typology of defects in the different graphene samples [25]. In particular, the *I*(D)/*I*(D′) ratio exhibits its maximum value, around 13, for sp^3^-like defects, it decreases down to about 7 in the case of vacancies and it is minimum for edge defects (ca. 3.5). In our case the estimated *I*(D)/*I*(D′) value is around 4.4, indicating that defects in the material are reasonably ascribed to the boundaries.

### Gas-sensing study

In order to investigate the reliability of the fabrication process in terms of gas-sensing behaviour, two identical devices have been considered, labelled with A and B. As described below in the Experimental section, the sensing layer has a rectangular geometry: *l* = 206 μm, *w* = 5 μm (Figure 1) and *t* = 7.2 nm. The two devices have been tested towards 1 ppm of NO_2_, 250 ppm of NH_3_ and 10 ppm of CO in N_2_ atmosphere. An inert carrier gas rather than a more realistic environment (i.e., by employing synthetic air) was chosen because of the undesired influence of oxygen. The number of active sites on the graphene surface decreases due to binding of O_2_ molecules via weak physisorption [26]. This phenomenon tends to impair the quantitative study that will be carried out afterwards, leading to an underestimation of the amount of adsorbable analyte. However, it is important to underline that oxygen does neither actively and crucially affect the physics of the sensing process nor the performance of the devices, which is only worsened to a little extent.

**Figure 3:**
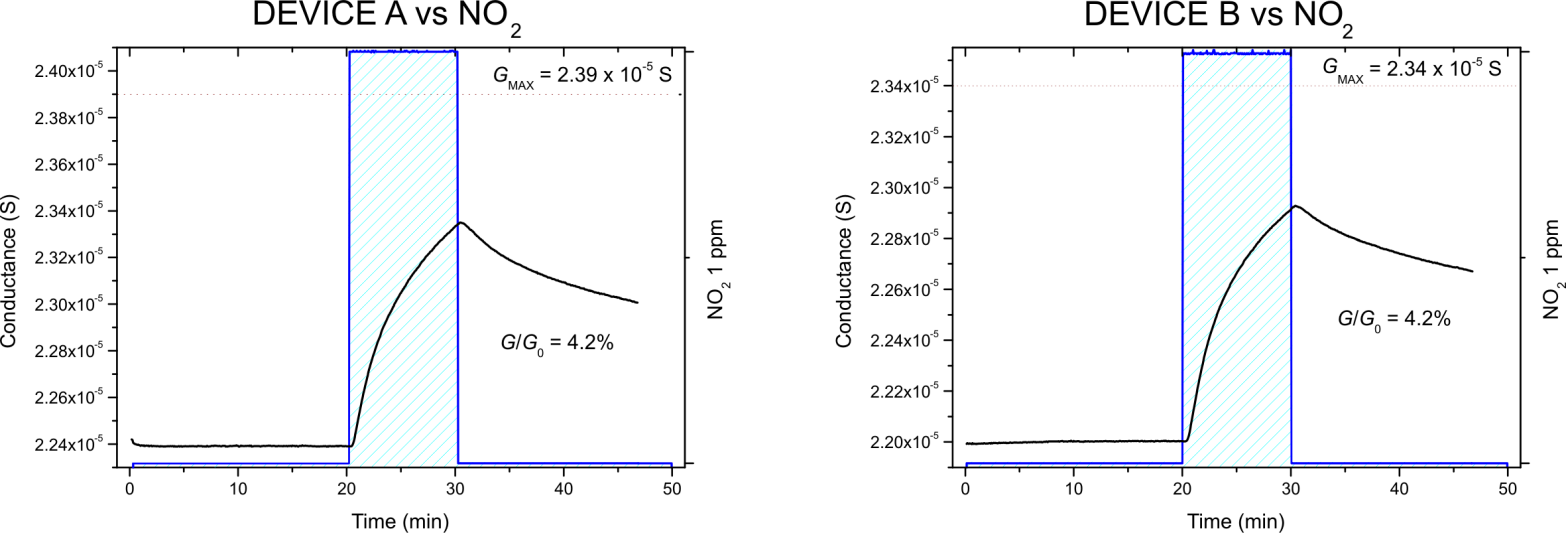
Dynamic response of devices A (left) and B (right) during the exposure to 1 ppm of NO_2_.

Figure 3 displays the dynamic response of the conductance of the devices towards 1 ppm of NO_2_. As can be observed, the base conductance values are comparable (*G*_A_ ≈ *G*_B_ ≈ 22 µS). During exposure, both chemiresistors exhibit an identical percentage variation of the conductance without reaching a plateau. In the figures the asymptotic values of conductance are also displayed. Such quantities have been evaluated by extrapolating the final conductance value by fitting each curve, within the time window of exposure to the analyte, with the exponential function


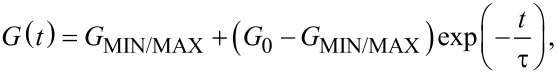


where *G*_MIN/MAX_ represents the asymptotic value of the conductance after exposure to the analyte. When the gas inlet is stopped, a slow decrease of the device conductance and thus an incomplete recovery follows. This is not surprising and can be explained in terms of interaction energy between graphene and analyte, as will be explained later. Since NO_2_ acts as an electron acceptor, during interaction the electronic charge is transferred from graphene to the analyte. Thus, the observed increase in the device conductance is naturally expected because of the interaction of the molecule with a p-type material.

**Figure 4:**
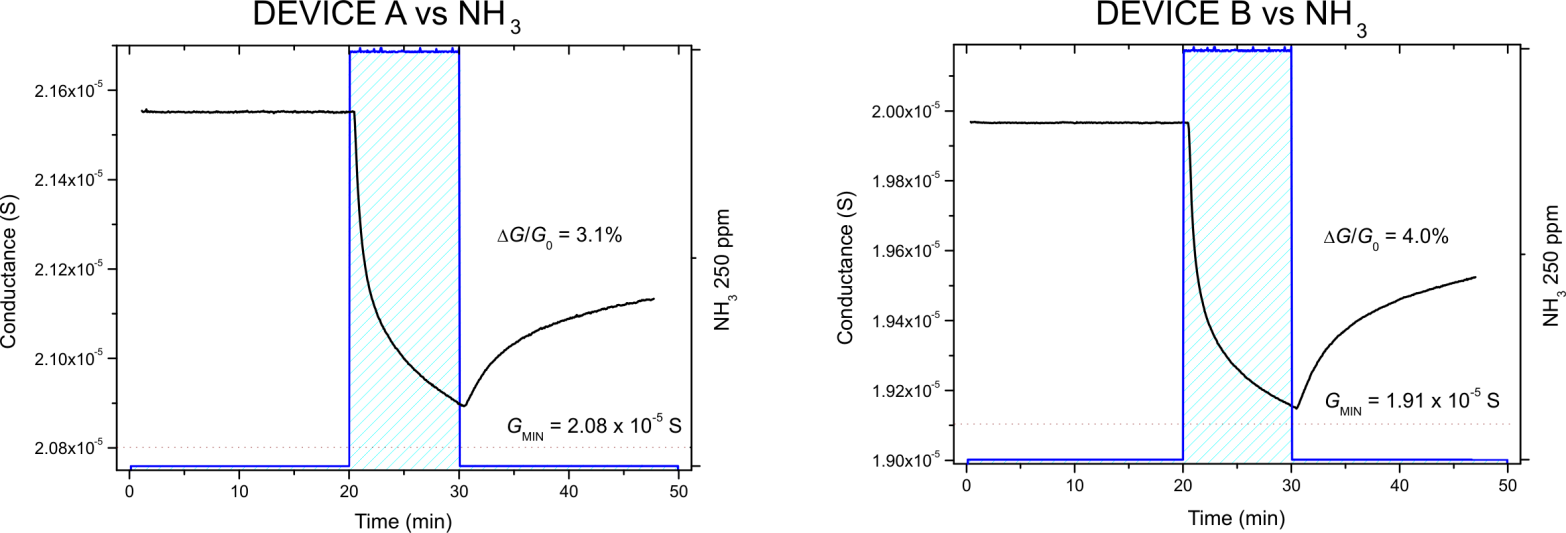
Dynamic response of devices A (left) and B (right) during the exposure to 250 ppm of NH_3_.

Subsequently, the devices have been exposed to NH_3_. Even in this case both chemiresistors exhibit a comparable percentage variation of the conductance, as well as similar kinetics (Figure 4). Because ammonia is an electron-donor analyte, the conductance show a decrease, further confirming the p-type nature of the synthesized graphene. At the end of the exposure to the analyte, the restoration of the initial conditions appears slow and incomplete.

Following the protocol described in the Experimental section, the devices have been finally exposed to 10 ppm of CO. The devices were completely unresponsive towards CO and therefore the measurements are not shown.

Apart from the direction of the conductance variation, the performances of the devices towards both NO_2_ and NH_3_, in terms of Δ*G*/*G*_0_ and response kinetics, appear almost comparable. In order to discriminate the differences in the graphene behaviour towards the two analytes at a deeper level, a reasonable description of the adsorption process should be provided. These mechanisms are conventionally well described by Arrhenius’ law and the adsorption energies can be derived from the characteristic time of the desorption curve at different temperatures [27–28]. However, the desorption observed in the examined devices appears to be a result of different concurring phenomena, all of which comparably contribute to the exponential behaviour of the curve with a different characteristic time.

Hereinafter, an alternative point of view is proposed, based on the modification of the electronic properties of the material due to the analyte adsorption. Similarly to Arrhenius' concept of activation energy, this approach still includes the fundamental assumption of the validity of Maxwell–Boltzmann statistics.

The absolute number of analyte molecules approaching the devices has been firstly estimated, referring to the responses reported in Figure 3 and Figure 4. Within the approximation of an isotropic, quasi-stationary regime in the testing chamber and schematizing the chemiresistors surface as a rectangle of length *l* and width *w*, the total flow (500 sccm) has been converted into a number of analyte molecules per unit time, 
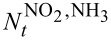
, by knowing the incoming concentrations (1 ppm for NO_2_ and 250 ppm for NH_3_). The total pressure in the testing chamber (*p*_TOT_ = 1 atm) has been taken into account. Furthermore, a uniform molecule density *d*_V_ = *N_t_*/*V*_c_ in the chamber (*V*_c_ = 0.4 dm^3^) has been assumed:


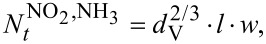



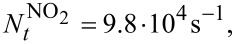



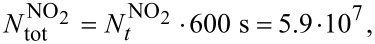


The effective number of colliding analyte molecules during the exposure in the time interval Δ*t* = 600 s has finally been evaluated as:


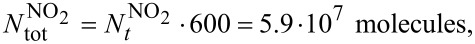



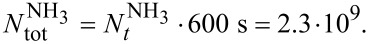


It is worth noting that the ration of these quantities for NO_2_ and NH_3_ is 250^−2/3^. This factor will be used later to normalize the number of adsorbed molecules to the same amount of incoming analyte.

The initial conductance value *G*_0_ of the devices (lateral dimensions *l* and *w*, thickness *t*) obeys Ohm’s law:


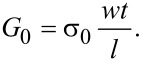


The conductivity can be expressed in terms of volumetric density and mobility of the charge carriers:


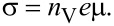


According to the mobility values reported in literature for CVD graphene (between 10^2^ and 10^3^ cm^2^/V·s), in the calculations, a carrier mobility of μ ≈ 1000 cm^2^/V·s has been assumed [29–31]. During the exposure, the number of carriers per unit surface has changed by the fraction Δ*G*/*G*_0_ in 10 min. This quantity is linked to the number of adsorbed molecules via the virtual charge-transfer amount resulting from the interaction between graphene and analyte molecules, Δ*q*. Hence, the effective number of analyte-induced charge carriers *N*_S_ onto the devices surface can be expressed as


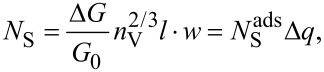


where 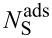 is the number of analyte molecules adsorbed on the surface of the sensing film. A charge transfer of −0.19*e* for NO_2_ and 0.02*e* for NH_3_ has been assumed, according to the theoretical results found by Zhang et al. on NO_2_ and NH_3_ adsorption on pristine graphene [32]. This choice has been made conforming to the results of the morphological characterizations, which attest a low defect density of the material and absence of substitutional impurities, so the material can be reasonably assumed as pristine.

The degree of adsorption, α, is the ratio between the total number of adsorbed molecules and the total number of colliding analyte molecules during exposure:


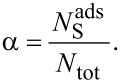


For both examined devices a different behaviour can be distinguished for the two analytes, in particular α_NO2_ ≈ 5α_NH3_ has been found, showing a predisposition of the sensing material towards NO_2_ detection (Table 1 and Table 2).

**Table 1:** Summary of the relevant quantities descending from the interaction between graphene (devices A and B) and 1 ppm of NO_2_ during 10 min of exposure, calculated based on the responses reported in Figure 3.

	1 ppm NO_2_					
	Δ*G*/*G*_0_ (%)	*N*_S_|*e*|	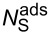	α	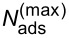	*E*_int_ (eV)

device A	4.2	1.7·10^6^	9.1·10^6^	0.16	1.5·10^7^	−0.413 ± 0.001
device B	4.2	1.6·10^6^	8.0·10^6^	0.14	1.3·10^7^	−0.409 ± 0.001

**Table 2:** Summary of the relevant quantities descending from the interaction between graphene (devices A and B) and 250 ppm of NH_3_ during 10 min of exposure, calculated based on the responses reported in Figure 4.

	250 ppm NH_3_					
	Δ*G*/*G*_0_ (%)	*N*_S_|*e*|	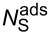	α	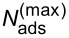	*E*_int_ (eV)

device A	3.1	1.3·10^6^	6.3·10^7^	0.03	6.7·10^7^	−0.358 (error < 10^−4^)
device B	4.0	1.5·10^6^	7.6·10^7^	0.03	8.3·10^7^	−0.363 (error < 10^−4^)

Finally, the characteristic energies related to the interaction between graphene and NO_2_/NH_3_ have been estimated. These parameters refer to the maximum number of molecules that can be adsorbed on the sensing strip. 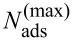 has been obtained by extrapolating the maximum “asymptotic” increase/decrease of the conductance and converting it into the maximum amount of adsorbed molecules on the sensing strip by using the previous calculations. As far as NH_3_ is concerned, 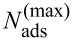 has been multiplied by the adjusting factor 250^−2/3^ in order to normalize the concentrations for both analytes.

Analogously to the conventional treatment of doped semiconductors, after having estimated the maximum number of adsorbed analyte molecules, they have been assimilated as punctual impurities in the crystal lattice. Herein the Maxwell–Boltzmann approximation for the statistical description of charge carriers can be justified by virtue of the semi-classical regime of the system, with low induced charge density, of the order of 10^−2^ with respect to that of the “undoped” material (≈10^12^ cm^−2^, which is an usual value for pristine graphene [33]) and a temperature sufficiently far from the quantum regime, 0 K. Indeed, when exp(*E*/*k*_B_T) >> 1, it is possible to write


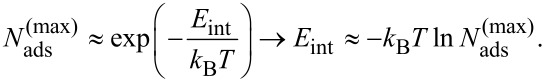


In particular, the estimated characteristic energies for NO_2_, both for devices A and B (Table 1), appear comparable to theoretical results regarding adsorption processes on graphene [31,34–35]. The calculated values also justify the slow and incomplete desorption of molecules, the thermal energy of which (*k*_B_*T* ≈ 25 meV) is not sufficient to “escape” from the potential well associated to the adsorption.

The primary quantities (Δ*G*/*G*_0_, *N*_S_, 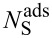, α, 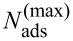 and *E*_int_) calculated for devices A and B, for NO_2_ and NH_3_, are summarized in Table 1 and Table 2. As can be seen, these values are very similar for the two devices.

The presented quantitative model, assuming a static configuration of the system, is not fully representative of the complex charge carrier dynamics due to the adsorption of gas molecules on the graphene surface. Herein, additional mechanisms that affect the conductance behaviour, such as induced charge propagation within the layers of a single graphene grain, as well as the transfer through the edges of adjacent grains, are not taken into account. However, the utilized fabrication technique, which confers a remarkable control on the sensing film geometry, makes a simplifying schematization possible, so as to reasonably quantify the affinity between graphene and the different analytes at room temperature via the as-defined interaction energy *E*_int_ and the degree of adsorption α.

The drawback of slow and incomplete recovery in devices working at room temperature is well-known in the literature [13,36–37]. This disadvantage can be overcome by following different strategies, such as the introduction of a heater or a UV light source on board, able to restore the basic device conditions at the end of exposure to analyte [9–13]. Another possibility is to record the signal derivative of conductance [8] with a differentiator operational amplifier. In a CMOS-compatible process, all these devices could be directly fabricated on the same chip at micrometre-size. It is finally worth mentioning that, once proven the reliability of this process, it paves the way for the creation of a sensor array, able to provide selective responses towards the analytes. In addition, the possibility of miniaturizing such an array goes in the direction of creating a portable electronic nose.

## Conclusion

We have shown the potential of a transfer-free deposition technique for the fabrication of graphene-based gas sensors by a process that is fully compatible with silicon electronics. The synthesis technique has proven to be highly replicable, as identical devices exhibit comparable performances. Notwithstanding a change of sign in the conductance variation, the chemiresistive devices have exhibited analogous responses towards NO_2_ and NH_3_, besides being totally insensitive to CO. The similarity in the behaviour towards NO_2_ and NH_3_ has been studied in depth by evaluating the characteristic energies associated to the different analytes by following an unconventional approach, focusing on the modification of the electronic properties of the sensing material due to the analyte adsorption rather than the kinetics of the surface reactions. From this analysis a predisposition of transfer-free CVD graphene towards NO_2_ detection has clearly emerged. The remarkable control on the sensing film geometry, which is specific to the presented technique, enables a quantification of the interaction energy and the degree of adsorption of NO_2_ and NH_3_ at room temperature employing a simple descriptive model, the results of which are coherent with those reported in other theoretical works related to gas-adsorption processes on graphene.

The recovery time for these devices is typically extremely long, affecting the time stability of the base conductance as well as the sensing performances. The direct-growth approach for the synthesis of graphene opens a promising path towards the integration in electronic devices and the employment of additional technologies suitable for both speeding up the gas desorption and overcoming the abovementioned drawbacks.

## Experimental

4″ Si wafers coated by 90 nm thick thermal SiO_2_ layer have been employed for the synthesis. A thin film of Mo has been sputtered on top of it, starting from a pure (99.95%) Mo target. The metal film thickness can be made as thin as 50 nm without any segregation at the graphene CVD temperature (≈1000 °C), because of the higher melting point of Mo (2623 °C) compared to that of other conventional catalysts such as Cu (1085 °C) or Ni (1455 °C). Moreover, this allows a pre-patterning the film for the selective growth of graphene down to micrometre-size dimensions. A photo-lithographic process, combined with SF_6_ dry etching, has been used to shape the Mo in the desired form.

Graphene layers have then been grown on pre-patterned Mo/SiO_2_/Si substrate by means of AIXTRON BlackMagic Pro equipment, setting the temperature at 1000 °C, the pressure at 25 mbar and using Ar/H_2_/CH_4_ as feedstock. At the end of the CVD growth, the Mo has been selectively etched away underneath graphene employing phosphoric acid. Due to anchoring at the sides of the patterned catalyst, the graphene layer sticks directly to the underlaying SiO_2_. Cr/Au metal contacts have finally been patterned by a lift-off process to design a four-point probe structure. The final chemiresistive devices consist of a material strip 206 µm long and 5 µm wide, placed between two couples of metal electrodes (Figure 1).

Raman analyses have been performed at different points of the sensing strips by means of Renishaw InVia Reflex spectrometer, at λ = 514 nm and 20 mW incident laser power, in backscattering configuration.

The chemiresistors have been tested in a Gas Sensor Characterization System (GSCS, Kenosistec equipment) consisting of a stainless steel testing chamber (*V* = 0.4 dm^3^), placed in a thermostatic box. Precise amounts of analyte were let into the chamber via a system of programmable mass flow controllers (MKS) and electro-pneumatic valves. Relative humidity can be adjusted as required through a water bubbler placed in a thermostatic bath. The bias voltage is provided by TTi QL355T Precision Power Supply.

The following measurement protocol, schematized into three runs for a total duration of 50 min, has been employed: (1) 20 min carrier gas flow (baseline), (2) 10 min exposure to the analytes at the chosen concentrations, (3) 20 min carrier gas flow (recovery). The measurements have been carried out in inert N_2_ environment, keeping the total flow at 500 sccm, at atmospheric pressure, relative humidity set at 50%, *T* = 25 °C. The devices have been kept at 1 V DC bias voltage. The sampling rate for the acquisition of the conductance values has been set to 0.25 Hz. The dynamic response has been quantified as the percentage variation of the conductance:


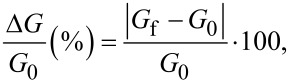


where *G*_0_ is the unperturbed conductance value and *G*_f_ is the conductance value at the end of the exposure.
